# Supplementary material for: Temporal proteomic profiling of postnatal human cortical development
Source: Transl Psychiatry. 2018 Dec 5;8:267. doi: 10.1038/s41398-018-0306-4 (PMC6281671; doi:10.1038/s41398-018-0306-4)
Supplement: Supplementary file 1 — Figure S1-11 [file 41398_2018_306_MOESM1_ESM.pdf]

SUPPLEMENTAL FIGURES

Title: Temporal proteomic profiling of postnatal human cortical development

Authors: Michael S. Breen[1,2,3†*], Sureyya Ozcan[4†], Jordan M. Ramsey[4], Zichen Wang[5], Avi Ma'ayan[5], Nitin Rustogi[4], Michael G. Gottschalk[4,6], Maree J. Webster[7], Cynthia Shannon Weickert[8,9,10], Joseph D. Buxbaum[1,2,3‡], Sabine Bahn[4‡]

Affiliations: [1]Department of Psychiatry, [2]Department of Genetics and Genomic Sciences, [3]Seaver Autism Center for Research and Treatment, Icahn School of Medicine at Mount Sinai, New York, New York, 10029, USA; [4]Department of Chemical Engineering and Biotechnology, University of Cambridge, Cambridge, United Kingdom, CB3 0AS; [5]Department of Pharmacological Sciences, Mount Sinai Center for Bioinformatics, BD2K-LINCS Data Coordination and Integration Center, Knowledge Management Center for Illuminating the Druggable Genome (KMC-IDG), Icahn School of Medicine at Mount Sinai, New York, New York, 10029, USA; [6]Department of Psychiatry and Psychotherapy, Medical Center, Faculty of Medicine, University of Freiburg, Germany; [7]Stanley Medical Research Institute, Laboratory of Brain Research, Rockville, Maryland, 20815, USA; [8]Schizophrenia Research Laboratory, Neuroscience Research Australia, Randwick NSW 2031, Australia; [9]School of Psychiatry, Faculty of Medicine, University of New South Wales, Sydney, NSW, 2052, Australia; [10]Department of Neuroscience & Physiology, Upstate Medical University, Syracuse, New York, 13210, USA.

Correspondence to michael.breen@mssm.edu

This file contains **Supplemental Figures 1-11.** In brief:

Supplementary Figure 1. Quality control of proteomic and transcriptomic data.
Supplementary Figure 2. Results for proteins with low limits of detection.
Supplementary Figure 3. Results of individual age-related proteins and RNAs.
Supplementary Figure 4. Weighted correlation networks for proteome and transcriptome.
Supplementary Figure 5. Scatterplots of protein module eigengenes and age.
Supplementary Figure 6. Characterization of protein modules M3 and M5 (not age-related).
Supplementary Figure 7. Protein-protein interaction networks for protein modules M1, M2 and M4.
Supplementary Figure 8. Summary of cell type specific markers in proteome and transcriptome.
Supplementary Figure 9. Correspondence between transcriptome data and BrainSpan data.
Supplementary Figure 10. Age-related transcriptome modules and functional annotation.
Supplementary Figure 11. Module preservation analysis (proteome vs. transcriptome).

Figure S1

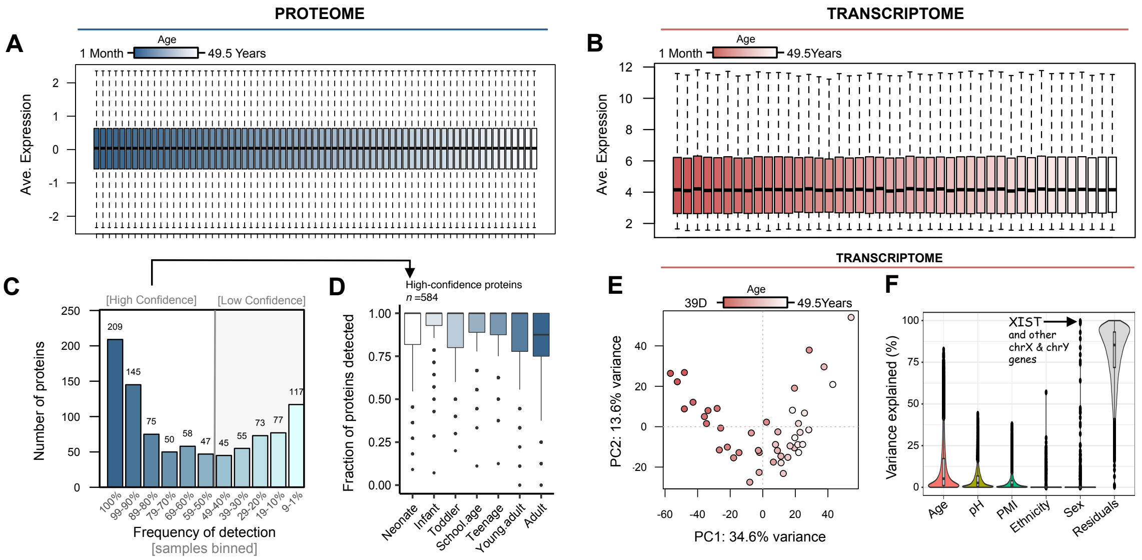

Figure S1. Quality control of proteome and transcriptome data. (**A and B**) Normalized boxplots of proteome (blue) and transcriptome (red) samples. (**C**) Histogram of detected protein abundance across bins (%) of the total sample. High-confidence proteins are defined as being detected across > 50% of the sample. (**D**) Levels of protein detection/quantification across each developmental stage. (**E**) Principal component analysis on global normalized transcriptome data, samples are shaded by age. (**F**) variancePartition mixed linear model analysis of global gene expression identifies age as a leading trait explaining the most amount of observed gene expression variability.

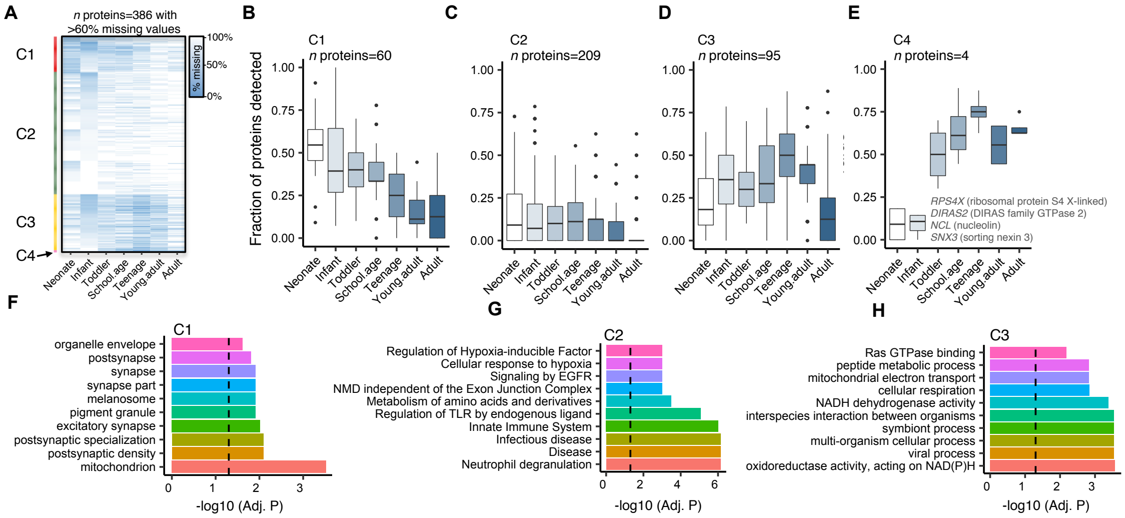

Supplemental Figure 2. Protein detection and quantification for 386 proteins with missing data. (a) Unsupervised hierarchical clustering (Pearson's distance and wards clustering) was used to identify clusters of proteins, which grouped together by observed patterns of protein quantification/detection ratios across all developmental stages, and four clusters were identified. (b) Cluster 1 (C1) contained 60 proteins, for which ratios of protein detection were biased towards early development. (c) Cluster 2 (C2) contained 209 proteins, for which ratios of protein detection were not biased towards any developmental stage. (d) Cluster 3 (C3) contained 95 proteins, for which ratios of protein detection were biased towards teenage years. (e) Cluster 4 (C4) contained 4 proteins, which are listed at the inset of panel e, for which ratios of protein detection were biased towards adulthood. Functional enrichment analysis of (f) C1 (g), C2, and (h) C3 were carried out to characterize the top 10 most significant biological process, molecular factors and/or pathways associated with each cluster. C4 was not functionally annotated due to too few proteins within the cluster.

Figure S3

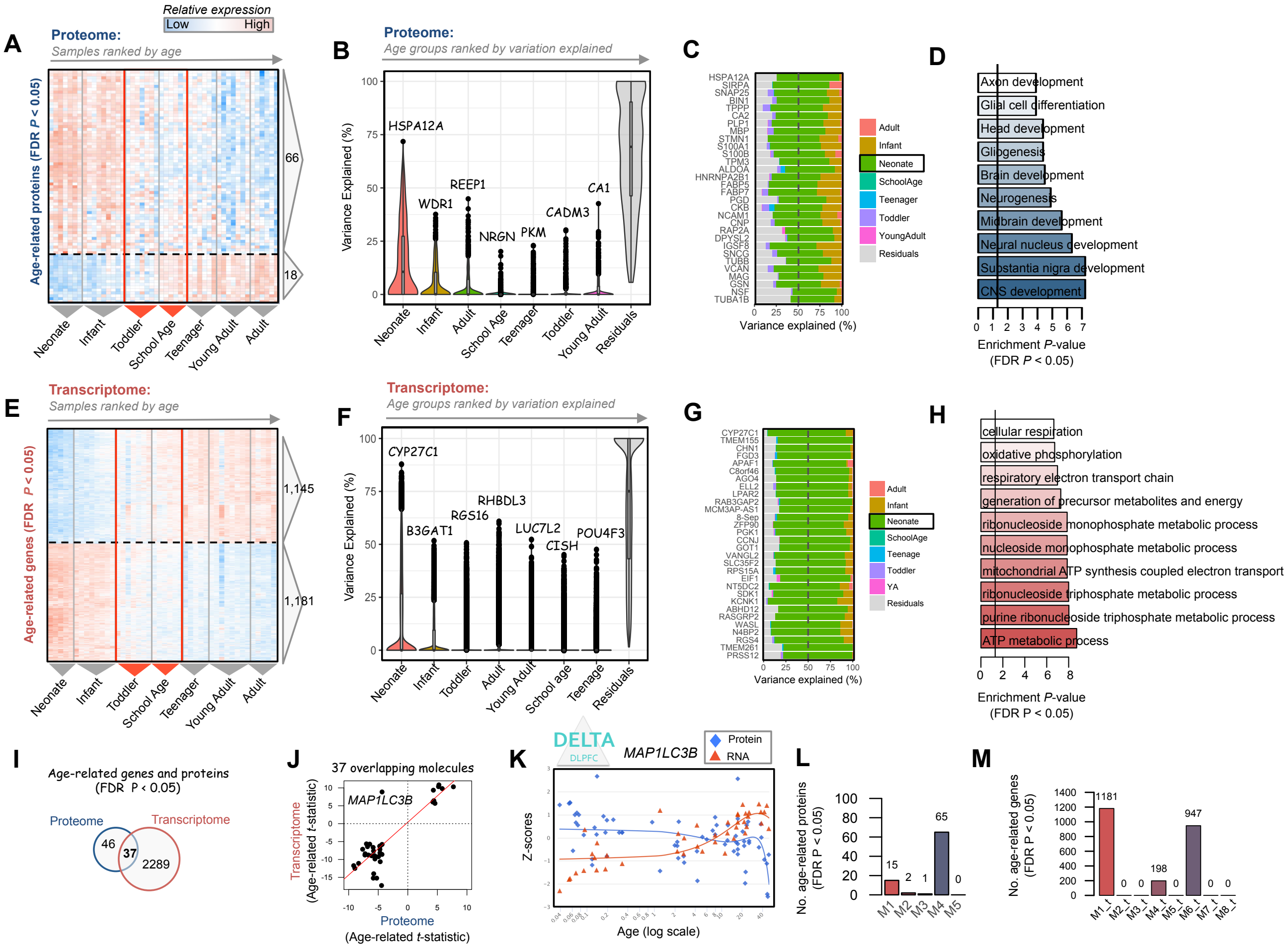

Figure S3. Linear modelling of proteome and transcriptome data. (**A**) Heatmap of significant age-related proteins (FDR *P* < 0.05). (**B**) variancePartition analysis of global protein abundance by age group identifies the neonate group explaining the most amount of observed protein variability across development. The top proteins for each age group-related are displayed. (**C and D**) Top 30 neonate explained proteins (>50%) and their (**D**) top 10 functional annotations according to gene ontology biological processes. (**E**) Heatmap of significant age-related genes (FDR *P* < 0.05). (**F**) variancePartition analysis of global transcriptome data by age group identifies the neonate group explaining the most amount of observed gene expression variability across development. The top genes for each age group-related are displayed. (**G and H**) Top 30 neonate explained genes (>10%) and their (**H**) top 10 functional annotations according to gene ontology biological processes. (**I**) Overlap of age-related proteins and genes. (**J**) Correspondence of age-related *t*-statistics for the 35 overlapping proteins and RNAs. (**K**) Interactive, visualization using DELTA in DLPFC for *MAP1LC3B* gene and protein expression. **(L and M)** Module enrichment of significant age-related (**L**) proteins and (**M**) RNAs.

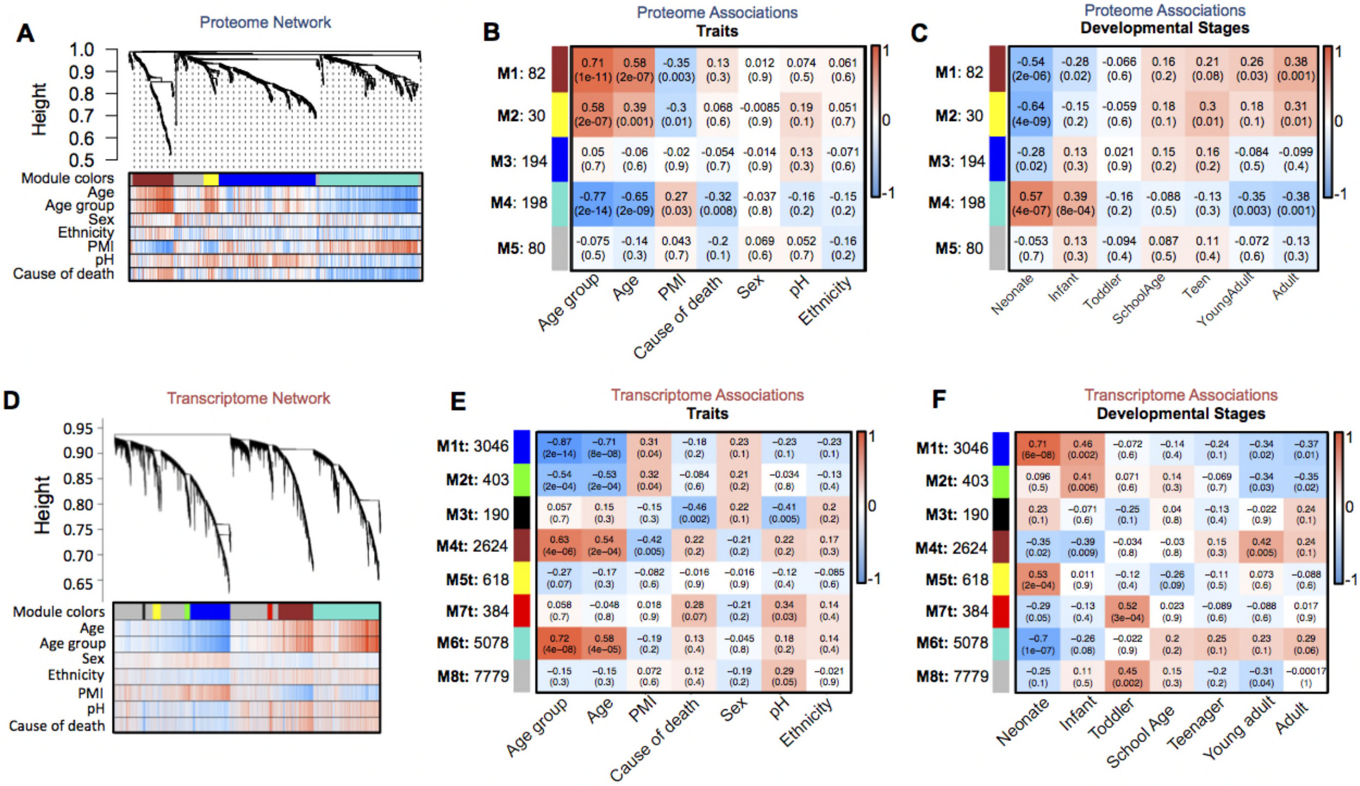

Figure S4. Proteome and transcriptome modular organization in developing DLPFC. (**A**) Protein dendrogram and identified modules. Each gene module is indicated by the color bar below the protein hierarchical cluster tree and subsequent bars indicate protein-trait relationships; red indicates positive association, blue indicates negative association. (**B and C**) Proteome module eigengene (ME) correlations with collected traits/covariates as well as (**C**) developmental age groups. For each cell, Pearson's correlation coefficient (*r*) is displayed as top value and the corresponding Student asymptotic *P*-value are displayed bottom. (**D**) In a similar fashion, the constructed gene dendrogram with identified modules, and trait relationships are displayed. (**E and F**) Transcriptome ME correlations with collected traits/covariates as well as (**F**) developmental age groups.

Figure S5

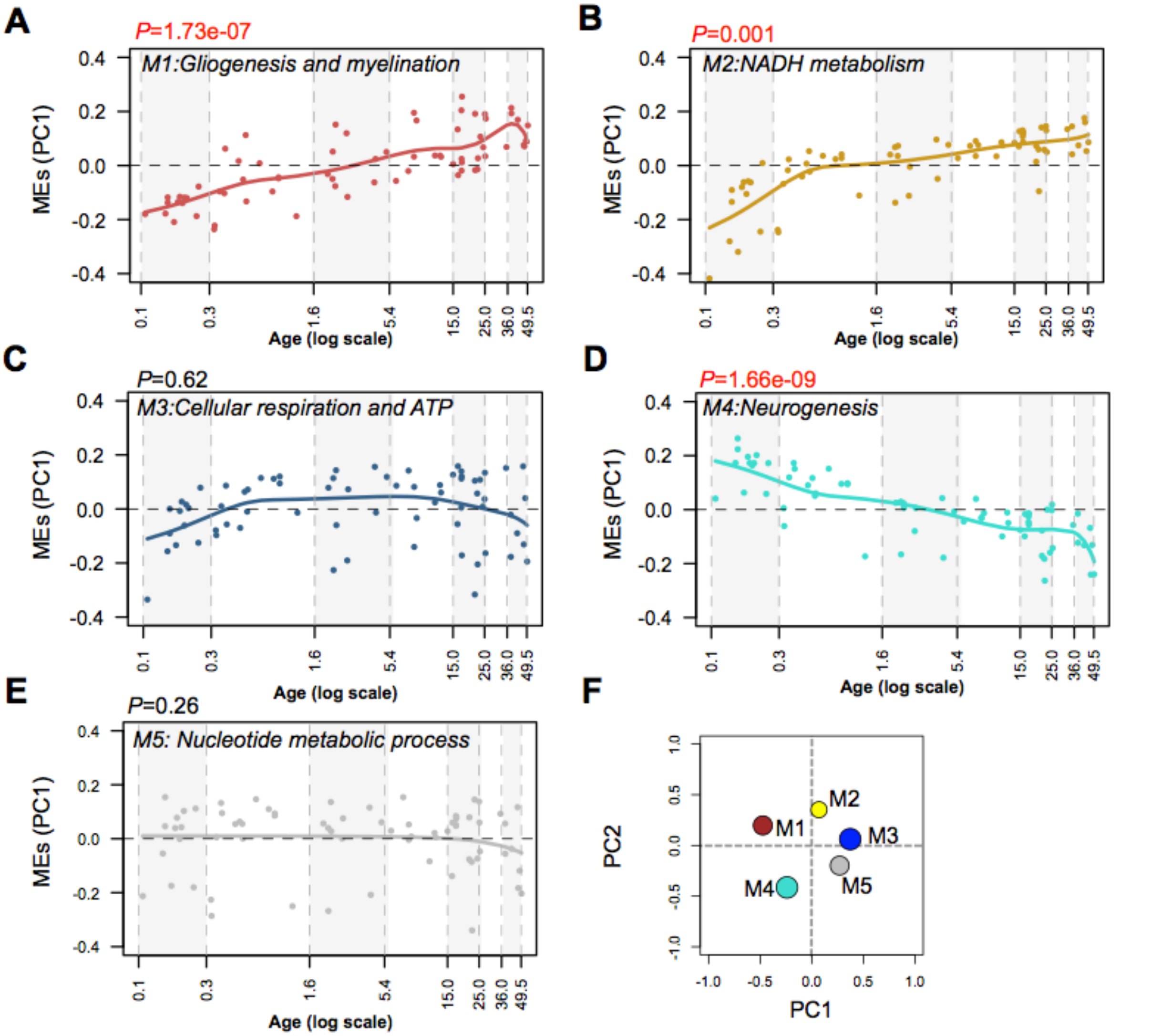

Figure S5. Scatterplots of protein module eigengenes (MEs) and postnatal age. (**A-E**) Samples are shaded by developmental stage and smoothed splines (knot=4) were used to plot best fit lines, corresponding *P*-values are displayed. (**F**) Principal component analysis of all protein MEs separates modules by function and age significance.

Figure S6

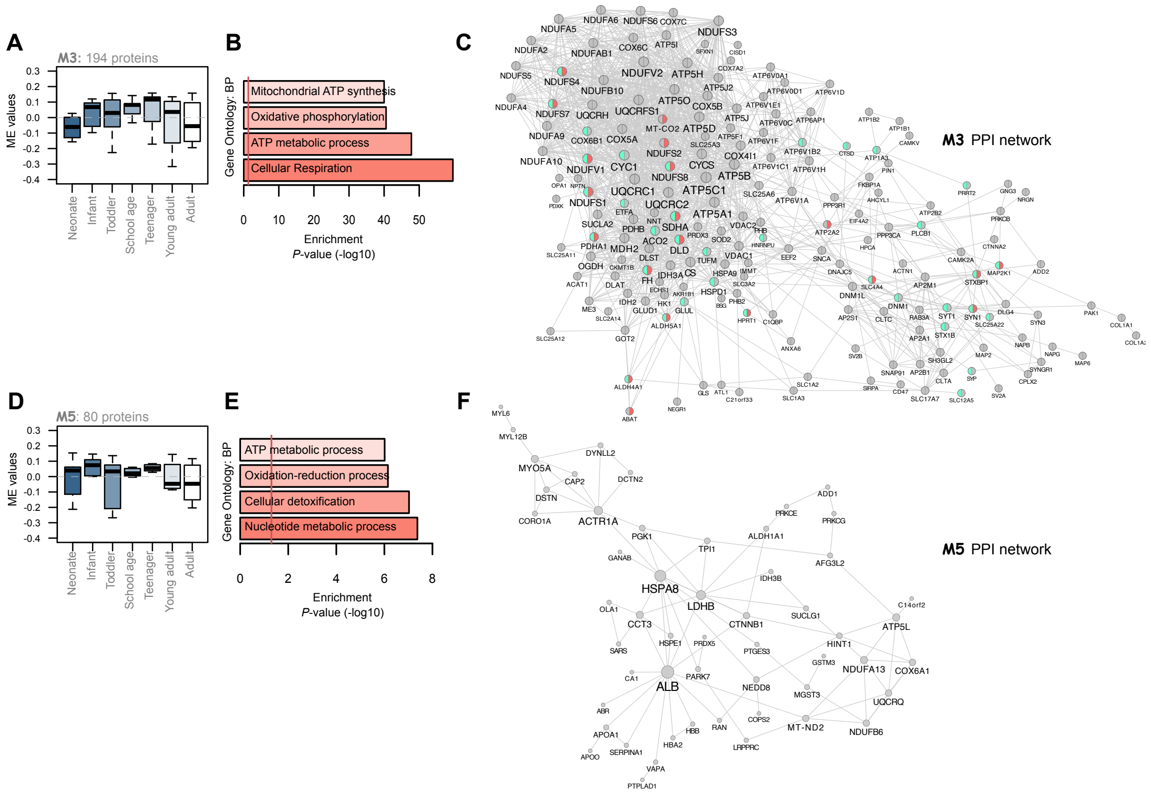

Figure S6. Module characteristics for modules M3 and M5. (**A**) Module eigengene (ME) expression for protein module M3 throughout developmental stages. (**B and C**) Functional annotation (GO: biological processes) and (**C**) direct protein-protein interaction (PPI) network topology for module M3. Nodes are split into halves; red indicates intellectual disability risk loci and blue indicates developmental delay risk loci. (**D**) ME expression is displayed for protein module M5 throughout developmental stages. (**E and F**) Functional annotation (GO: biological processes) and (**F**) direct PPI network topology for module M5. Direct PPIs were collected from the STRING database (https://string-db.org/) and imported into Cytoscape for visualization. Larger nodes indicate greater degree of connectivity (i.e. hub genes).

Figure S7

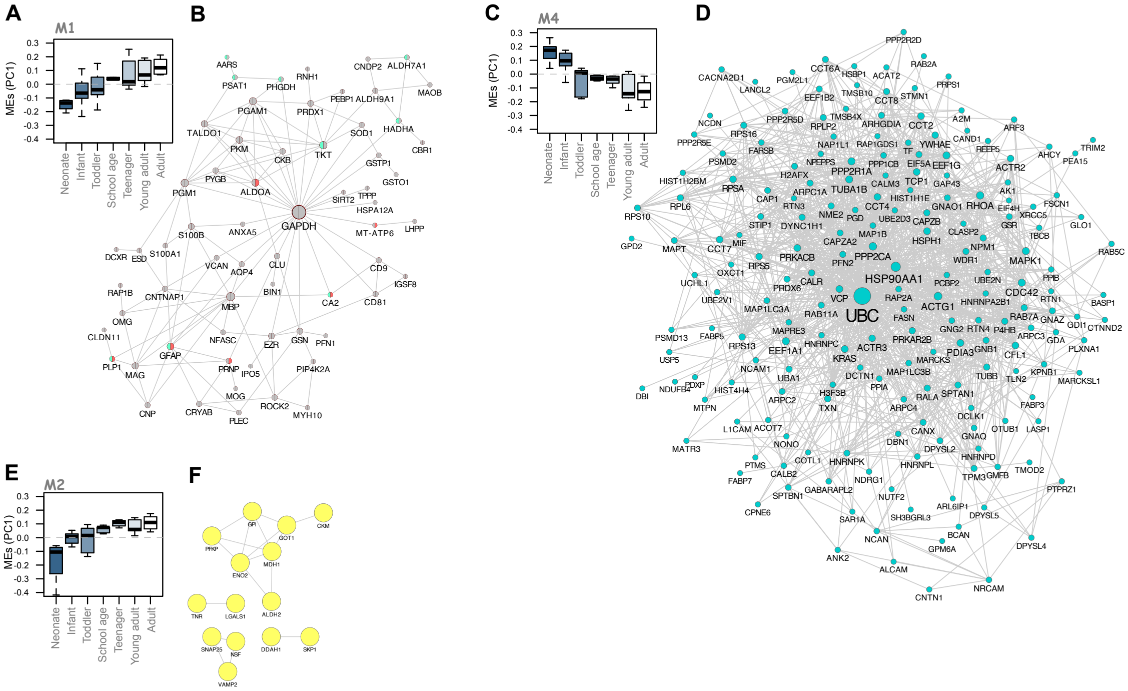

Figure S7. Module characteristics for modules M1, M2 and M4. (**A and B**) Module eigengene (ME) expression for protein module M1 throughout developmental stages along with (**B**) direct protein-protein interaction (PPI) network topology. Nodes are split into halves; red indicates intellectual disability risk loci and blue indicates developmental delay risk loci. (**C and D**) ME expression and (**D**) direct PPI networks are plotted for module M2. (**E and F**) ME expression and (**F**) direct PPI networks are plotted for module M4. Direct PPIs were collected from the STRING database (https://string-db.org/) and imported into Cytoscape for visualization. Larger nodes indicate greater degree of connectivity (i.e. hub genes).

Figure S8

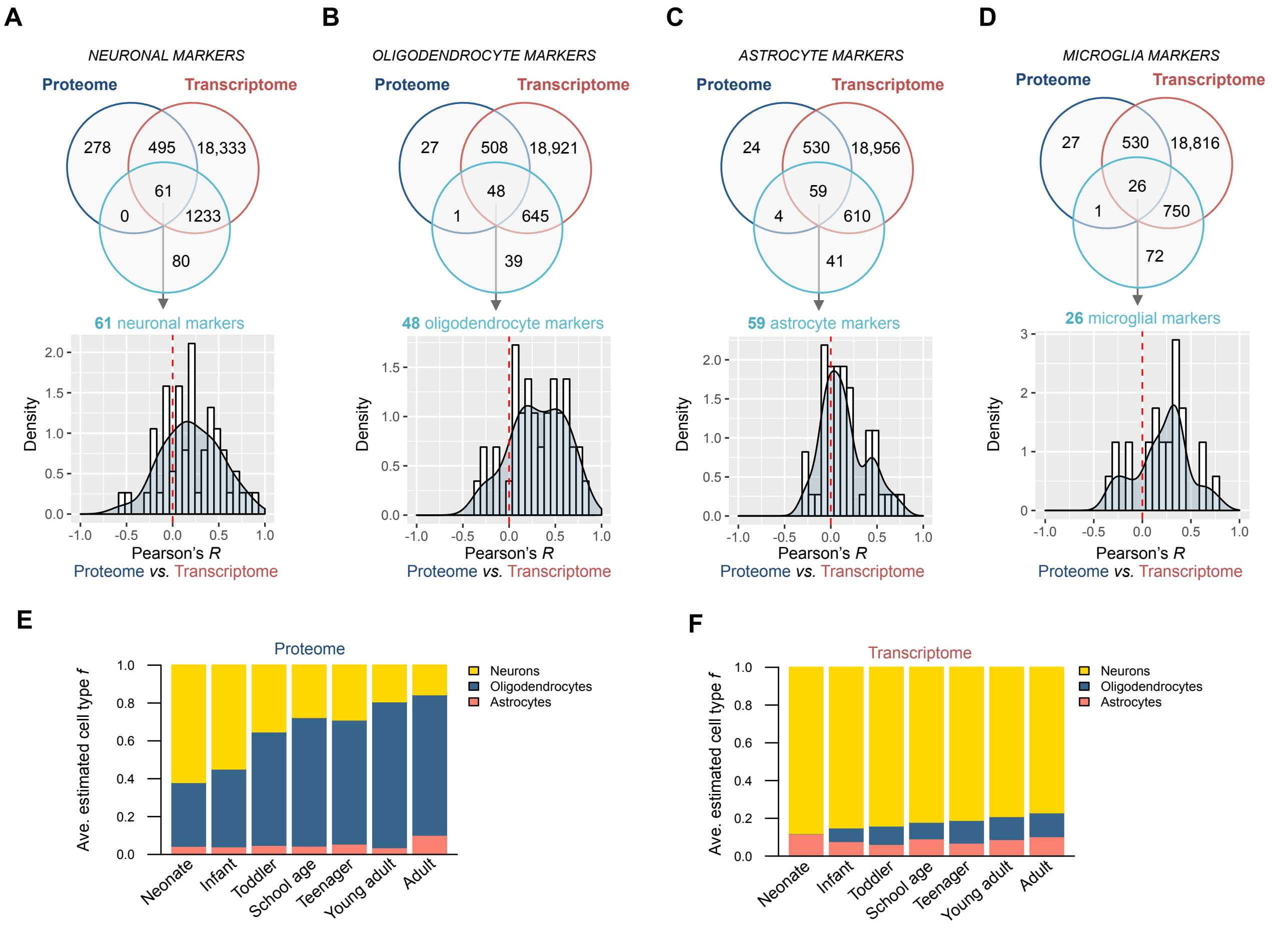

Figure S8. Concordance between protein and RNA cell type specific markers. (**A-D**) Overlap of (**A**) neuronal markers, (**B**) oligodendrocyte markers, (**C**) astrocyte markers and (**D**) microglial markers followed by the correlation coefficient between RNA and protein expression levels. **Figure information:** Cell type markers were selected from previous RNA-sequencing (Zhang et al., 2014; Zeisel et al., 2015) and proteomic (Sharma et al., 2015) investigations (*see Methods*). These lists were pooled for each cell type, labeled as cell type specific if they were detected across two or more of these studies, and then overlapped with the detected proteome and transcriptome. (**E and F**) Average Cibersort cell type deconvoultion estimates (y-axis) for (**E**) proteomic data and (**F**) transcriptomic data pooled by developmental stages (x-axis).

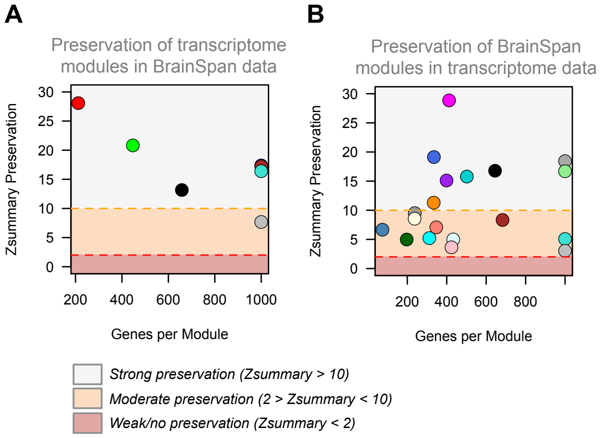

C

BrainSpan ID (*N*=17)	Age
18_DFC_m_4	4 months
19_DFC_m_4	4 months
19.5_DFC_m_10	10 months
20_DFC_y_1	1 yr.
21_DFC_y_2	2 yrs.
22_DFC_y_3	3 yrs.
23_DFC_y_4	4 yrs.
24_DFC_y_8	8 yrs.
25_DFC_y_8	8 yrs.
26_DFC_y_11	11 yrs.
27_DFC_y_13	13 yrs.
28_DFC_y_18	18 yrs.
30_DFC_y_21	21 yrs.
31_DFC_y_30	30 yrs.
32_DFC_y_36	36 yrs.
33_DFC_y_37	37 yrs.
34_DFC_y_40	40 yrs.

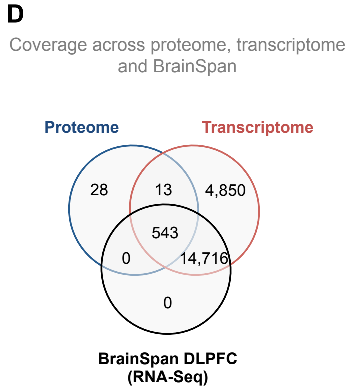

Figure S9. Preservation of transcriptome-based modules in postnatal DLPFC BrainSpan data. (A) Preservation of transcriptome-based modules in postnatal DLPFC BrainSpan data (http://www.brainspan.org/). **(B)** Preservation of BrainSpan modules in the current transcriptome data. Note that the current study measures the temporal transcriptome on 44 postnatal DLPFC samples, compared to 17 DLPFC postnatal samples in BrainSpan. **(C)** Sample characteristics for the 17 BrainSpan samples used to support module reproducibility. A $Z_{summary}$ preservation score < 2 indicates no evidence of preservation, 2 < $Z_{summary}$ < 10 implies weak preservation and $Z_{summary}$ > 10 suggests strong preservation. **(D)** Level of detection across technologies, including brain proteome, microarray transcriptome and BrainSpan RNA-sequencing. Commonly used RNA-sequencing filtering techniques reduced the number of genes to 15,272.

Figure S10

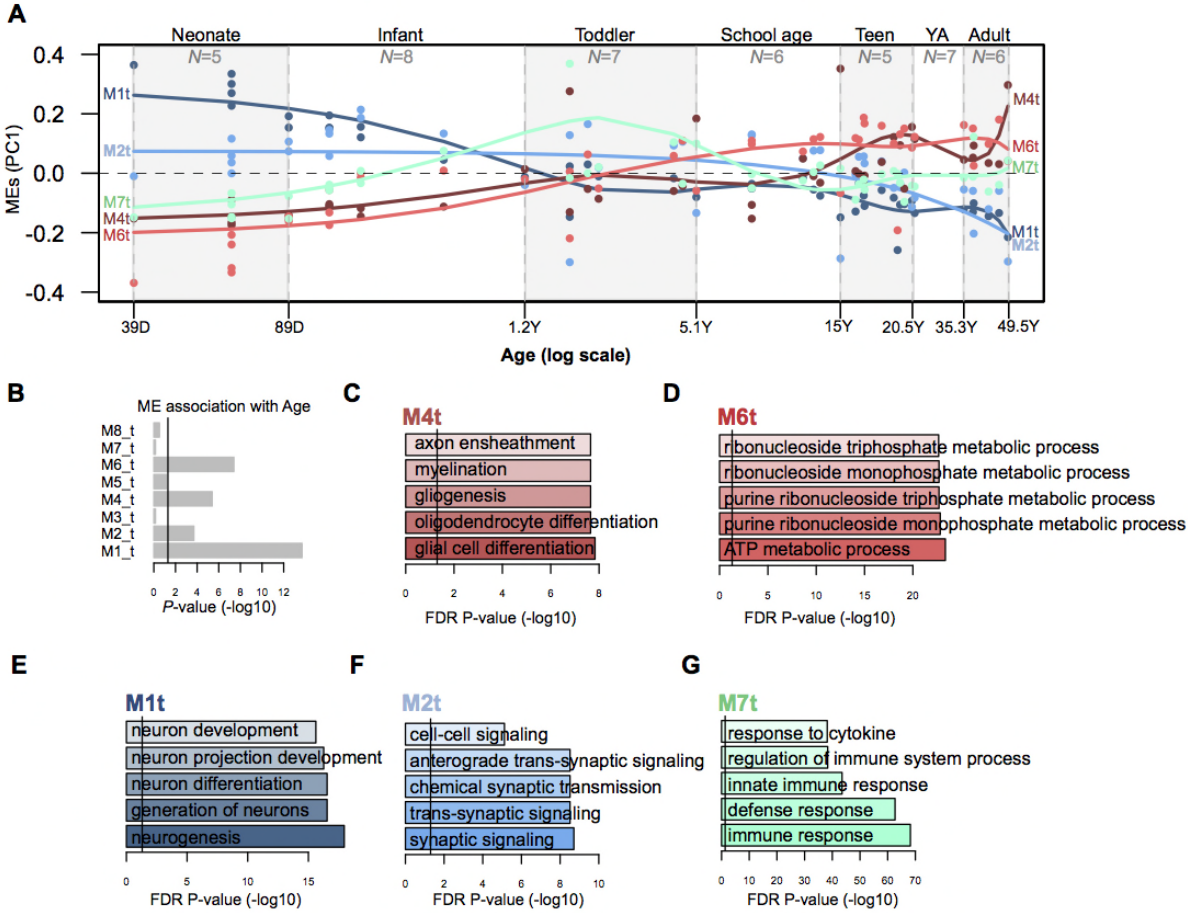

Figure S10. Postnatal age-related transcriptome modules. (A) Module eigengene (ME) postnatal developmental trajectories of four age-related transcriptome modules and one transcriptome module significantly associated with the developmental stage of toddlers (1.2-5.1 years). Developmental stages are shaded (grey/white) and labeled, and the number of samples measured for each stage are displayed above. (**B**) Results from transcriptome ME associations with postnatal human age. (**C-G**) Functional annotation for the top age-related transcriptome modules, x-axis indicates false discovery corrected –log10 *P*-value significance.

Figure S11

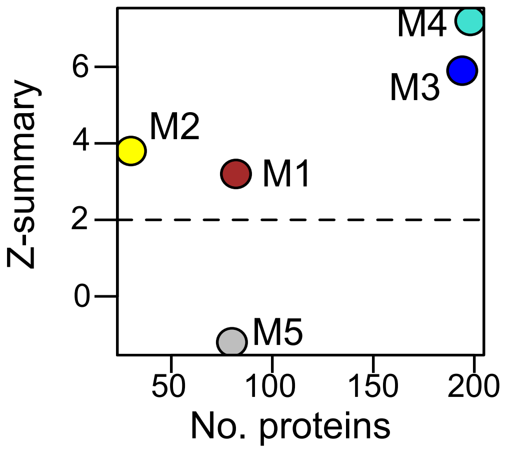

Figure S11. Module preservation analysis. Preservation of protein co-regulation patterns at the RNA level; $Z_{summary}$ <2 indicates no preservation, 2< $Z_{summary}$ <10 indicates weak preservation.
